# Natural Killer T-Cell Agonist α-Galactosylceramide and PD-1 Blockade Synergize to Reduce Tumor Development in a Preclinical Model of Colon Cancer

**DOI:** 10.3389/fimmu.2020.581301

**Published:** 2020-10-20

**Authors:** Ying Wang, Madhura S. Bhave, Hideo Yagita, Susanna L. Cardell

**Affiliations:** ^1^Department of Microbiology and Immunology, Institute of Biomedicine, University of Gothenburg, Gothenburg, Sweden; ^2^Department of Immunology, Juntendo University School of Medicine, Bunkyo-ku, Japan

**Keywords:** NKT (natural killer T) cells, tumor, intestine, PD-1, check-point blockade, alpha-galacosylceramide

## Abstract

Murine and human invariant natural killer T (iNKT) lymphocytes are activated by α-galactosylceramide (α-GalCer) presented on CD1d. α-GalCer was first described as a lipid that had strong anti-metastatic effects in a mouse melanoma model, and it has subsequently been shown to induce efficient iNKT cell dependent tumor immunity in several tumor models. We have shown that α-GalCer treatment leads to a weak reduction of polyp burden in the autochthonous *Apc^Min/+^* mouse model for human colon cancer, however this treatment resulted in upregulation of the inhibitory receptor PD-1 on iNKT cells. While anti-PD-1 treatment can prevent immune-suppression in other cancer types, human colon cancer is generally resistant to this treatment. Here we have used the *Apc^Min/+^* model to investigate whether a combined treatment with α-GalCer and PD-1 blockade results in improved effects on polyp development. We find that PD-1 expression was high on T cells in polyps and lamina propria (LP) of *Apc^Min/+^* mice compared to polyp free ^*Apc+/+*^ littermates. Anti-PD-1 treatment alone promoted Tbet expression in iNKT cells and CD4 T cells, but did not significantly reduce polyp numbers. However, the combined treatment with anti-PD-1 and α-GalCer had synergistic effects, resulting in highly significant reduction of polyp numbers in the small and large intestine. Addition of PD-1 blockade to α-GalCer treatment prevented loss of iNKT cells that were skewed towards a TH1-like iNKT1 phenotype specifically in polyps. It also resulted in TH1 skewing and increased granzyme B expression of CD4 T cells. Taken together this demonstrates that a combination of immune stimulation targeting iNKT cells and checkpoint blockade may be a promising approach to develop for improved tumor immunotherapy.

## Introduction

The CD1d-restricted natural killer T (NKT) cells are an unconventional T cell subset with immunoregulatory potential. NKT cells regulate a diverse range of immune reactions and inflammation, including in infections and autoimmune disease, tumor immunity and adipose tissue homeostasis (1–4). Invariant NKT (iNKT) cells make up an evolutionarily conserved subset of NKT cells that expresses a semi-invariant T cell receptor (TCR) with an invariant TCR α-chain rearrangement (Vα24-Jα18 in humans and Vα14-Jα18 in mice), paired with a limited number of TCR β-chains (5). The potent anti-tumor potential of iNKT cells was first discovered when it was shown that iNKT cells were responsible for the strong effect of α-galactosylceramide (α-GalCer) preventing liver metastasis in a mouse model (6, 7). α-GalCer is presented on the MHC class I-like molecule CD1d and activates virtually all iNKT cells in both mice and humans (8). The invariant nature of the iNKT cell TCR, and its non-polymorphic restriction element CD1d makes iNKT cells attractive targets for immunotherapy. Numerous studies have been performed in which α-GalCer has been successfully used to modulate immune responses in experimental tumor models (9–11). This has led to clinical trials that apply iNKT cell activation to enhance anti-tumor immune activity in cancer patients (8, 12–15).

Colorectal cancer (CRC) is the third most common cause of cancer death and the fourth most commonly diagnosed cancer worldwide (16). Tumor infiltrating lymphocytes are a strong predictor of relapse and overall survival in CRC (17). More specifically, IFN-γ dominated TH1 type immune profiles of tumor infiltrating cells were associated with improved prognosis while TH17 responses correlated with poor prognosis (18). The immune contexture offers an improved predictor of patient survival compared to microsatellite staging of CRC patients, as some microsatellite stable (MSS) tumors show high immune cell infiltration (19). Presence of TH1/CTL immune cell infiltrates and expression of IFN-γ and the TH1 transcription factor TBET occurs predominantly in microsatellite instable (MSI) colorectal cancers, however, this is associated with high expression of checkpoint inhibitors on infiltrating immune cells (20). This lends an explanation as to why CRC tumors with TH1/CTL immune infiltrates are not naturally eliminated, but also as to why patients with MSI tumors respond to immune checkpoint blockade treatment. Blockade of immune inhibition such as the PD-1/PD-L1 and CTLA-4 pathways can enhance anti-tumor responses and antibody blockade of these pathways has become an established cancer treatment (21, 22). Checkpoint blockade with antibodies targeting CTLA-4 or the PD-1 pathway is primarily effective in the 10% to 15% subset of CRC patients with MSI tumors, while most patients have MSS tumors (23, 24). The failure of immune checkpoint therapy in MSS CRC (25) highlights the need to explore treatments that combine checkpoint therapy with other immune activating therapies to improve disease outcome.

*Apc^Min/+^* mice are a well-established animal model for CRC and reflect early events in the disease (26–28). Loss of the tumor suppressor gene adenomatous polyposis coli (*APC*) is regarded as an initiating event and is found in around 80% of sporadic CRC tumors. *APC* is also the gene mutated in familial adenomatous polyposis (FAP), an inherited form of CRC. The *Apc^Min/+^* mouse model carries a heterozygous mutation in the *Apc* gene, resulting in multiple intestinal neoplasias (Min) (26, 29). *Apc^Min/+^* mice are a model for MSS as polyps in *Apc^Min/+^* mice show loss of heterozygosity, i. e. they have lost the expression of the wild type *Apc* allele, but are generally genomically stable (30, 31). As in CRC, the immune system plays an important role in regulating tumor growth in *Apc^Min/+^* mice, and the model is frequently used for mechanistic studies of tumor immunopathogenesis in intestinal cancer (28). We have shown that treatment with α-GalCer only has a weak tumor suppressive effect in *Apc^Min/+^* mice (32). The effect may have been limited by the induction of anergy in iNKT cells by repeated α-GalCer treatment, characterized upregulated expression of PD-1 on iNKT cells in treated mice (32–34). We therefore hypothesized that addition of PD-1 blockade might improve treatment with the iNKT cell agonist α-GalCer and enhance anti-tumor activities. In this study, we have performed preclinical immunotherapeutic studies in the *Apc^Min/+^* mouse model and demonstrated that the combined treatment with α-GalCer and PD-1 blockade increased the activation of iNKT cells, enhanced the anti-tumor response and highly significantly and synergistically reduced intestinal tumor development in small and large intestines.

## Materials and Methods

### Mouse Strains and Breeding

The *Apc^Min/+^* breeding (26) on the C57BL/6 genetic background was maintained by crossing male *Apc^Min/+^* mice with female *Apc^+/+^* mice. Both male and female *Apc^Min/+^* mice were used and we did not observe any difference in tumor numbers between the genders (data not shown). All mice were bred and maintained at the department of Experimental Biomedicine, University of Gothenburg. All animal experiments were approved by the regional animal ethics board of Gothenburg (ethical permit number 1554/18).

### *In Vivo* Treatment With PD-1 Blockade and α-GalCer

*Apc^Min/+^* mice were treated from 12 weeks of age. Mice were intraperitoneally (i.p.) administered with 0.25 mg anti-PD-1 antibody RMP1-14 (35) in PBS twice a week. α-GalCer (Avanti^®^, Polar Lipids Inc.) was administered weekly i. p., 4µg in 200μl of PBS solution. Lyophilized α-GalCer had been dissolved in PBS with 5.6% sucrose, 0.75% L-histidine and 0.5% Tween-20. Rat IgG was used as control and injected in an identical manner.

### Tumor Counting and Scoring

Mice were sacrificed at 15 weeks of age. The intestines were flushed with phosphate buffered saline (PBS) from both sides using blunt end gavage needles to remove fecal material, and were then cut open longitudinally. Tumors were counted and scored by size (<3mm or ≥3mm). In our animal facility, at 15 weeks of age an average of around 20 polyps are seen in the small intestine (penetrance, 100%; typical range, 5–35) and an average of 2 to 3 polyps are found in colon (penetrance, 75–90%; typical range, 0–5) (36).

### Lymphocyte Preparation

Spleen, mesenteric lymph nodes (MLN), polyp-free lamina propria (LP) and polyp tissue were collected from treated *Apc^Min/+^* mice. Single cell suspensions from spleen and MLN were prepared by forcing the organs though nylon mesh using a syringe plunger. LP lymphocytes and tumor-infiltrating lymphocytes were isolated from the SI after removal of Peyer’s patches. The intestine tissue was dissected into polyp and unaffected tissue to be processed separately. Unaffected tissue was cut into small pieces. Polyp and unaffected tissues were dissociated with Lamina Propria Dissociation Kit (Miltenyi Biotech, Bergisch Gladbach, Germany). Undigested tissue was removed by filtration and live lymphocytes counted using trypan blue dead cell exclusion solution (Gibco).

### Flow Cytometry

Tissues were processed, single cell suspensions were prepared and cells were stained. Fc block (Clone: 2.4G2) and vital dye (Live/Dead Fixable Aqua Dead Cell Stain, Invitrogen) were included in all the staining panels. Cells were stained with fluorescence-labeled antibodies obtained from BD Biosciences, eBioscience, and Biolegend. Intracellular staining and fixation were performed using the Foxp3/Transcription Factor Staining Buffer Set (Invitrogen™ eBioscience™). The following antibodies (clones) were used: CD3 (17A2), CD4 (RMA4-5), CD8 (53–6.7), CD11c (N418), CD11b (M1/70), CD19 (1D3), CD25 (PC61), CD44 (IM7), CD45 (30-F11), CD69 (H1.2F3), CD206 (C068C2), TCRβ (H57-597), ST2 (RMST2-33), KLRG1 (2F1), Ly6C (HK1.4), Ly6G (RB6-8C5), F4/80 (BM8), PD-L1 (MIH5), IFN-γ (XMG1.2), GZMB (NGZB), Ki67 (B56), FoxP3 (FJK-16s), T-bet (4B10), GATA3 (TWAJ), RORγt (Q31-378), NOS2 (CXNFT). Gating for the different immune cell populations were performed as shown in **Supplementary Figures 1 A, B**. BV-421 labeled CD1d-tetramers loaded with α-GalCer (PBS57) were kindly provided by the NIH Tetramer Facility. Stained samples were acquired on an LSR-II flow cytometer (BD Bioscience) and the results analyzed using FlowJo software (Tree Star Inc.) using the gating strategies shown in **Supplementary Figure 1**.

### *In Vitro* Stimulation

Lymphocytes were isolated from each organ as described above and 1 × 10^6^ cells were incubated in complete RPMI-1640 containing Brefeldin A (eBioscience™, Thermo Fisher Scientific). Following 3 to 4 h of stimulation with Cell Stimulation Cocktail (eBioscience™, Thermo Fisher Scientific) containing phorbol 12-myristate 13-acetate (PMA) and ionomycin at 37°C, cells were harvested and intracellular IFN-γ and granzyme B (GZMB) were detected with flow cytometry.

### Statistical Analyses

Calculation of statistical significance was performed using nonparametric Mann–Whitney test or Kruskal-Wallis test followed by Dunn’s multiple comparison post-test. P values of < 0.05 were considered significant. Statistical analyses were performed on Prism GraphPad 8 (GraphPad Software, La Jolla, CA). Results are presented as mean ± SD.

## Results

### PD-1 Was Highly Expressed on T Cells in Intestinal Lamina Propria of *Apc^Min/+^* Mice

**Figure 1:**
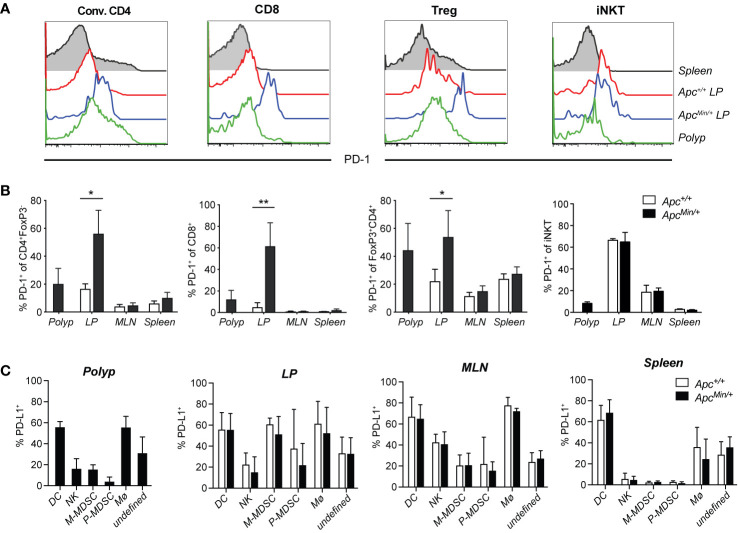
PD-1 and PD-L1 expression in tissues of *Apc^Min/+^* mice. *Apc^Min/+^* and *Apc^+/+^* littermate mice were sacrificed at 15 weeks of age. Cells were isolated from polyps, polyp-free lamina propria (LP) tissue, mesenteric lymph nodes (MLN) and spleen. **(A)** Representative surface expression of PD-1 on conventional CD4^+^ (Conv. CD4, CD4^+^ Foxp3^−^) T cells, regulatory T cells (Treg, FoxP3^+^CD4^+^) and CD8^+^ T cells, determined with flow cytometry (n = 4–7). **(B)** Frequency of PD-1^+^ cells among Conv. CD4, Treg and CD8^+^ T cells in the indicated organs. **(C)** PD-L1 expression on innate immune cell populations in 15-week-old *Apc^Min/+^* and *Apc^+/+^* mice: dendritic cells (DC, Lin(CD3, CD19)^−^CD45^+^CD11c^hi^), natural killer cells (NK, Lin^-^CD45^+^CD11c^lo/−^NK1.1^+^), monocytic myeloid-derived suppressive cells (M-MDSC, Lin^−^CD45^+^CD11c^lo/−^NK1.1^−^Ly6C^hi^Ly6G^lo^), polymorphonuclear MDSC (P-MDSC, Lin^−^CD45^+^CD11c^lo/−^NK1.1^−^Ly6C^int^Ly6G^+^), macrophages (Mø, Lin^-^CD45^+^CD11c^lo/−^NK1.1^−^Ly6C^−^Ly6G^−^F4/80^+^), unknown population (Lin^−^CD45^+^CD11c^lo/−^NK1.1^−^ Ly6C^−^Ly6G^−^F4/80^−^). The flow cytometry gating and frequencies of these immune cell populations in polyps and lamina propria is shown in **Supplementary Figure 1**. Data are presented as mean ± SD of 4–7 **(B)** and 4–6 **(C)** mice per group. Mann–Whitney test was used for statistical analyses. *p < 0.05, **p < 0.01.

Expression of PD-1 on T cells represents an immune checkpoint that serves as a break on T cell function, and PD-1 is absent in resting T cells. It was initially found upregulated on activated T cell subsets, but PD-1 has also been found on exhausted T cells in chronic infection models, as well as on tumor-infiltrating T cells in different types of cancer. To determine if PD-1 is expressed on T cells in polyps or unaffected surrounding intestinal tissue of *Apc^Min/+^* mice, we compared T cells from *Apc^Min/+^* mice and littermate *Apc^+/+^* mice. Polyp and LP (regardless of genotype) harbored 10% to 15% conventional CD4 T cells (CD4^+^FoxP3^−^, here termed CD4 T cells), around 5% CD8 T cells and 0.4% iNKT cells, while the frequency of regulatory T cells (Treg, CD4^+^FoxP3^+^) was over 40% in polyp tissue and around 20% in LP (**Supplementary Figure 1C** and **Figure 3A**). PD-1 was found on around 20% of CD4 T cells, 10% of CD8 T cells and 40% of Treg in polyps. Further, PD-1 was markedly upregulated in unaffected intestinal lamina propria (LP) of *Apc^Min/+^* mice, where 50% to 60% of all T cells were positive compared to less than 20% T cells expressing PD-1 in LP of *Apc^+/+^* mice (**Figures 1A, B**). In contrast, in mesenteric lymph node (MLN) and splenic T cells, PD-1 expression was generally low and comparable in *Apc^Min/+^* and *Apc^+/+^* mice. As we have shown before, around 10% of polyp iNKT cells express PD-1, and 60% of LP iNKT cells express PD-1 in *Apc^Min/+^* and *Apc^+/+^* mice (36). This indicates that T cells in LP and polyps of *Apc^Min/+^* mice may be negatively regulated by PD-1, resulting in dampening of T cell activation.

PD-L1 (CD274, also called B7-H1), one of the ligands of PD-1, is highly expressed on many tumor cells, antigen presenting cells, epithelial cells, parenchymal cells and virus infected cells (37, 38). In contrast, the PD-L2 ligand is found exclusively on professional antigen presenting cells (37). Inflammatory signals induce the expression of PD-L1 on tumor cells, which can inhibit T cell tumor immunity, thus, PD-L1 is also successfully targeted in checkpoint blockade treatment (22). However, in CRC the expression of PD-L1 is primarily found on hematopoietic cells and tumor stroma, and not on tumor cells (20). We investigated the expression of PD-L1 on CD45^+^ (hematopoietic) cells isolated from different tissues (see gating and frequencies of innate immune cell populations in **Supplementary Figures 1B–C**), and also analyzed the expression on CD45^−^ (non-hematopoietic) cells in polyp and LP (**Figure 1C** and **Supplementary Figure 2**). In LP of both *Apc^Min/+^* and *Apc^+/+^* mice, more than 90% of PD-L1^+^ cells were CD45^+^, while in polyps around 80% of PD-L1 expressing cells were CD45^+^ (**Supplementary Figure 2B**). Among CD45^+^ cells, the highest frequencies of PD-L1^+^ cells were found among dendritic cells and macrophages in polyp and LP, and also among monocytic myeloid derived suppressor cells (M-MDSC) in LP (**Figure 1C**). However, when considering the relative size of populations, the by far most abundant PD-L1^+^ population in polyp and LP was a less well defined TCR^-^ CD19^−^ population that was also negative for F4/80, NK1.1 and Ly6G (the “undefined” population in **Figure 1C** and **Supplementary Figure 2B**, defined in **Supplementary Figure 1B**). This PD-L1^+^ population was heterogenous, as some of the cells showed (mostly non-overlapping) expression of CD11b and intermediate levels of CD11c and Ly6C (**Supplementary Figure 1B**, **Supplementary Figure 2B** and data not shown). Further analysis revealed that innate lymphoid cell (ILC) 2 cells were present at around 2% of CD45 cells in polyp and LP, while ILC3 cells were present at slightly higher frequencies in LP compared to polyp (6–8% versus 2%, respectively, **Supplementary Figure 2C**). ILC2 and ILC3 cells contributed a minor proportion to the “undefined” population, and while around 15% of polyp ILC2 cells expressed PD-L1, less than 5% of LP ILC2 and ILC3 cells displayed this marker in *Apc^Min/+^* and *Apc^+/+^* mice (**Supplementary Figures 2D–E**). Thus, there was significant PD-L1 expression on hematopoietic cells in polyp and LP of *Apc^Min/+^* mice, but here were no significant differences in PD-L1 expression of any tissue between *Apc^Min/+^* and *Apc^+/+^* mice. Taken together, this suggests that T cells that reside in LP and polyp of *Apc^Min/+^* mice could be controlled by the PD-1/PD-L1 pathway.

### α-GalCer Activation of iNKT Cells Combined With PD-1 Blockade Synergistically Reduced Intestinal Polyp Development in *Apc^Min/+^* Mice

**Figure 2:**
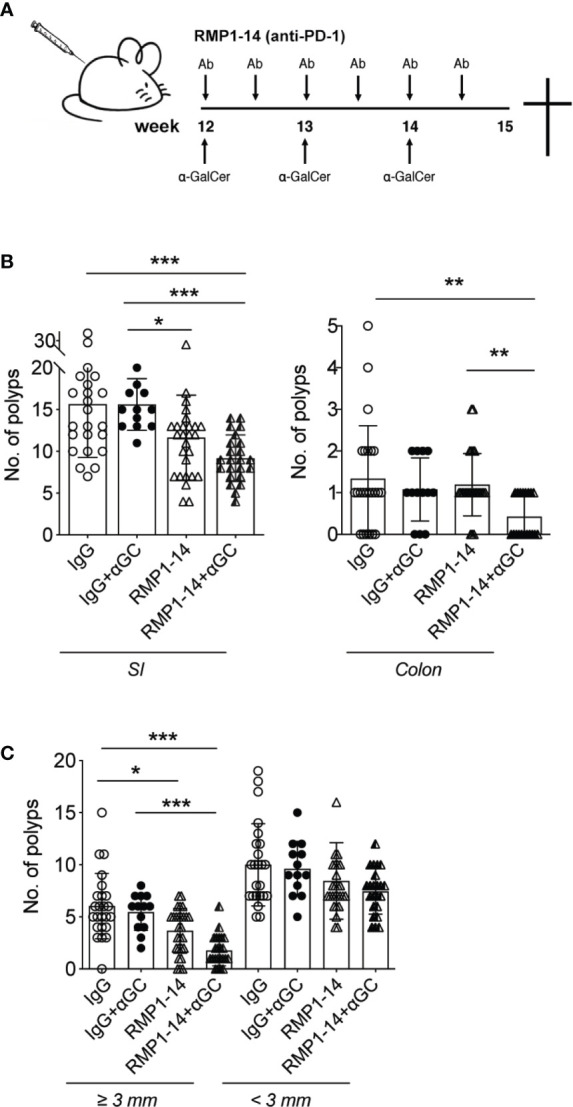
αGC and PD-1 blockade synergistically reduced intestinal tumor development. **(A)** Treatments were started when the *Apc^Min/+^* mice were 12 weeks of age. Mice were i.p. administered with 0.25 mg PD-1 antibody RMP1-14 twice per week, and/or weekly αGC treatments. Rat IgG was used as control antibody. **(B)** Treated mice were sacrificed at 15 weeks of age. Polyp numbers were counted in small intestine (SI, left graph) and colon (right graph). **(C)** Polyps were scored as large (diameter ≥ 3 mm) or small (diameter < 3 mm). Data were pooled from different sets of treatments and are presented as mean ± SD. Mice from each treatment group: IgG (n=23), IgG+α-GC (n=12), RMP1-14 (n=26), RMP1-14+αGC (n=23). Mann–Whitney test was used for statistical analyses. *p < 0.05, **p < 0.01, ***p < 0.001.

*Apc^Min/+^* mice develop polyps in both the small and large intestine. Early ileal microadenomas are found in one month old *Apc^Min/+^* mice (39), and at around 12 weeks of age, small polyp like lesions are visible on tissue sections by microscope (36). From this time point, polyps will grow and at 15 weeks of age an average of around 20 polyps are seen in the small intestine and an average of two to three polyps are found in colon (36). We recently demonstrated that a three-week treatment period of *Apc^Min/+^* mice with α-GalCer did not reduce polyp numbers, but only resulted in a reduction of polyp size in the small intestine (32). This was accompanied by systemically reduced iNKT cell frequencies, and an upregulated PD-1 expression on remaining iNKT cells, suggesting that iNKT cells in α-GalCer treated mice were anergic (33, 34). These findings, together with our demonstration that PD-1 was upregulated on T cells in the LP and polyps of *Apc^Min/+^* mice (**Figure 1**), supported our hypothesis that PD-1 blockade might improve treatment with the iNKT cell agonist α-GalCer and increase activation of both iNKT cells and conventional T cells in treated *Apc^Min/+^* mice. We therefore treated *Apc^Min/+^* mice for three weeks with the PD-1 antibody RMP1–14 in combination with α-GalCer, or each treatment alone. *Apc^Min/+^* mice were treated from 12 weeks of age following the schedule shown in **Figure 2A**, and sacrificed after three weeks. Compared to treatment with control antibody (rat IgG), treatment with α-GalCer alone did not result in reduction of polyp numbers, as shown before [**Figure 2B** and (32)]. Treatment with anti-PD-1 alone resulted in slightly reduced numbers and size of polyps in the small intestine. In contrast, treatment with the combination of anti-PD-1 and α-GalCer highly significantly and synergistically reduced polyp numbers in both the small intestine and colon (**Figure 2B**). Further, the number of large polyps in small intestine was strongly diminished after combination treatment (**Figure 2C**). Thus, the combination of PD-1 blockade and α-GalCer treatment effectively and synergistically reduced the development of polyps in *Apc^Min/+^* mice.

### Simultaneous PD-1 Blockade Prevented α-GalCer-Induced iNKT Cell Loss and Skewed Polyp iNKT Cells Toward an iNKT1 Phenotype

**Figure 3:**
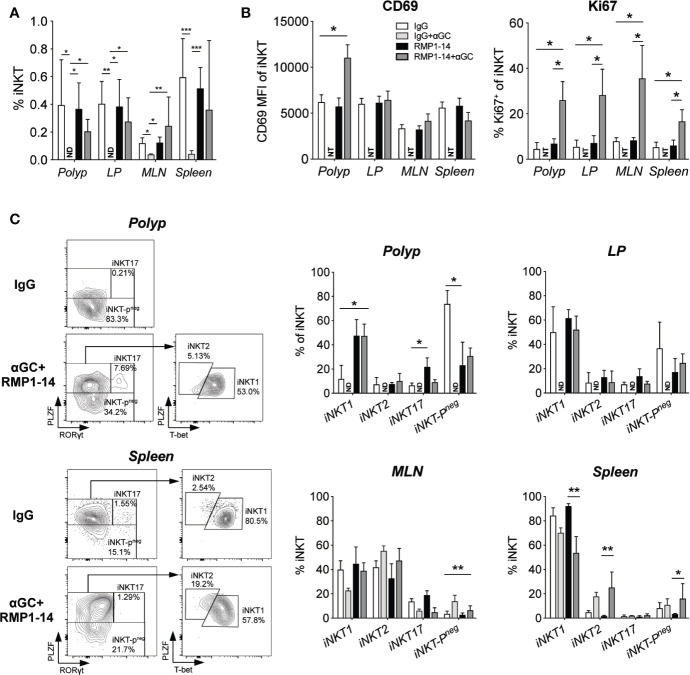
PD-1 blockade prevented αGalCer induced iNKT anergy and promoted an iNKT1 phenotype in *Apc^Min/+^* mice. *Apc^Min/+^* mice were treated according to the treatment schedule. Polyp, polyp-free LP tissue, MLN and spleen were harvested when the mice were sacrificed at 15 weeks of age. **(A)** The frequency of iNKT cells was determined by flow cytometry using α-GalCer (PBS57) loaded CD1d tetramer. ND = non-detectable. **(B)** The expression levels of the activation/tissue residence marker CD69 and the proliferation marker Ki67 on iNKT cells in different organs after treatments. Data were pooled from three experiments and are presented as mean ± SD of three mice per group. **(C)** iNKT cells were defined as iNKT1, iNKT2, iNKT17, and iNKT-P^neg^ according to the expression of PLZF, T-bet and RAR-related orphan receptor gamma t (RORγt). Representative stainings of polyp and splenic iNKT cells after vehicle (IgG) and combination (RMP1-14+αGC) treatment are shown (left). Bar graphs (right) summarize the proportions of iNKT1, iNKT2, iNKT17 and iNKT-P^neg^. Data are presented as mean ± SD of 5–8 mice per group. NT, not tested. Kruskal-Wallis test followed by Dunn’s multiple comparison post-test was used for statistical analyses. *p < 0.05, **p < 0.01, ***p < 0.001.

We next determined whether α-GalCer-induced loss of iNKT cells was prevented by the addition of PD-1 blockade in *Apc^Min/+^* mice. Confirming our previous study (32), we show that three weekly administrations of α-GalCer dramatically reduced the frequencies of iNKT cells in the tissues analyzed. After α-GalCer treatment, iNKT cells were not detected at all in polyps and LP (**Figure 3A**). In fact, there were too few iNKT cells remaining after treatment with α-GalCer alone to allow phenotypic analysis. In contrast, frequencies of iNKT cells were successfully maintained at normal levels in all tissues after treatment with the combination of anti–PD-1 and α-GalCer. The expression of the marker for proliferation Ki67 was significantly increased in iNKT cells in all organs analyzed, both as compared to mice treated with control IgG and anti–PD-1 alone (**Figure 3B**). Moreover, the combination treatment significantly increased the level of the early activation/tissue residence marker CD69 on iNKT cells in polyps, but not other tissues. Thus, PD-1 blockade prevented α-GalCer induced iNKT cell loss and resulted in increased iNKT cell proliferation and activation.

iNKT cells are characterized by the expression of the NKT cell master transcription factor promyelocytic leukemia zinc-finger (PLZF). Further, they can be divided into functionally different subsets based on the expression of transcription factors that determine cytokine secretion, in analogy to the CD4 TH cell subsets (40). iNKT1 cells express the TH1 transcription factor Tbet and produce IFN-γ, iNKT17 cells express RORγt and secrete IL-17, while iNKT2 cells that produce IL-4 are negative for Tbet and RORγt but have higher levels of PLZF. We have previously shown that polyp iNKT cells in *Apc^Min/+^* mice have a unique phenotype lacking PLZF expression (iNKT-P^neg^ cells in **Figure 3C**), and that they naturally increase polyp development by promoting Treg cells and suppressing TH1/CTL immunity (36). Therefore, considering the reduction of polyps that resulted from the combination treatment, we sought to determine if iNKT cells in polyps were altered. Indeed, treatment with anti-PD-1 and α-GalCer significantly increased iNKT cells in polyps with the iNKT1 transcription factor phenotype five-fold to around 50%, and reduced iNKT-P^neg^ cells from 70% to around 30%. Interestingly, this effect seems to result from the anti–PD-1 treatment, as similar effects were seen with this treatment alone. In contrast, there were no or only minor changes in iNKT cells in LP and MLN. Among splenic iNKT cells, there was a slight reduction of iNKT1 cells and increase of iNKT2 cells after treatment with α-GalCer with or without anti–PD-1. Thus, the combined α-GalCer and anti–PD-1 treatment led to increased activation and cycling of iNKT cells systemically, and a shift in transcription factor expression towards iNKT1 specifically in polyp iNKT cells, consistent with a local acquisition of anti-tumor function in polyp iNKT cells.

### α-GalCer Treatment Combined With PD-1 Blockade Promoted TH1 CD4 T Cells

**Figure 4:**
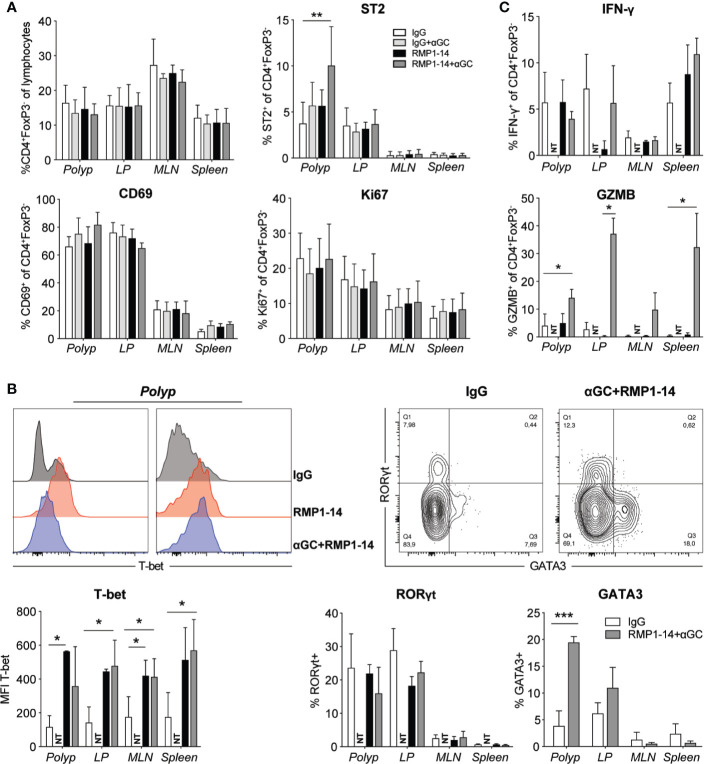
The PD-1 blockade and combination treatment promoted TH1 CD4^+^ T cells. *Apc^Min/+^* mice were treated according to the treatment schedule. Polyp, polyp-free LP, MLN and spleen were harvested when the mice were sacrificed at 15 weeks of age. **(A)** Frequency of Conv. CD4^+^ (CD4^+^FoxP3^−^) T cells among lymphocytes (upper left graph), and the expression of ST2, CD69 and Ki67 (as indicated) on Conv. CD4^+^ T cells in treated *Apc^Min/+^* mice. Data are presented as mean ± SD of 3 to 5 mice per group. **(B)** Representative stainings of T-bet, GATA3 and RORγt in Conv. CD4^+^ T cells (upper row) and summary bar graphs are shown. Data are presented as mean ± SD of 5 to 8 mice per group. **(C)** Lymphocytes were isolated from the indicated organs and were stimulated with PMA/ionomycin in the presence of brefeldin A for 3 to 4 h. Intracellular IFN-γ and granzyme B (GZMB) were detected with flow cytometry. Bar graphs show the frequency of IFN-γ^+^ and GZMB^+^ cells among Conv. CD4^+^ T cells. Data are presented as mean ± SD of four mice per group. NT, not tested. Kruskal-Wallis test followed by Dunn’s multiple comparison post-test was used for statistical analyses. *p < 0.05, **p < 0.01, ***p < 0.001.

**Figure 5:**
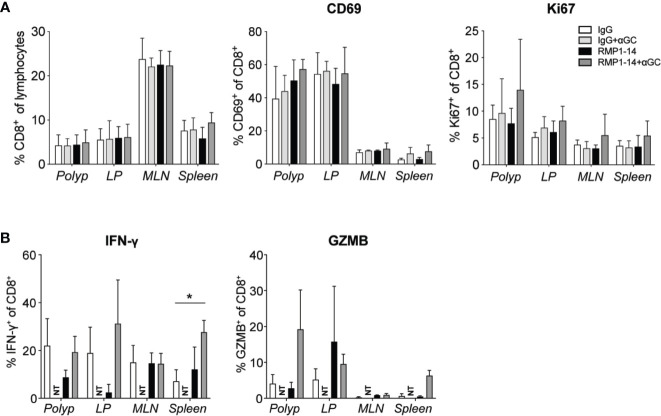
Analysis of CD8^+^ T cells after treatment. *Apc^Min/+^* mice were treated according to the treatment schedule. Polyp, polyp-free LP, MLN and spleen were harvested when the mice were sacrificed at 15 weeks of age. **(A)** Frequency of CD8^+^ T cells among lymphocytes (left), and the expression of CD69 and Ki67 (as indicated) on CD8^+^ T cells in treated *Apc^Min/+^* mice. Data are presented as mean ± SD of 3 to 5 mice per group. **(B)** Lymphocytes were isolated from the indicated organs and were stimulated with PMA/ionomycin with brefeldin A for 3 to 4 h. Intracellular IFN-γ and granzyme B (GZMB) were detected with flow cytometry. Bar graphs show the frequency of IFN-γ^+^ and GZMB^+^ cells among CD8^+^ T cells. Data are presented as mean ± SD of four mice per group. NT, not tested. Kruskal-Wallis test followed by Dunn’s multiple comparison post-test was used for statistical analyses. *p < 0.05.

We performed phenotypic analysis to determine whether there was an increased activation of polyp T cells associated with the reduced polyp burden after combination treatment (**Figures 4** and **5**). There was no difference in the frequencies of polyp infiltrating conventional CD4^+^ T cells or CD8^+^ T cells among lymphocytes after treatment (**Figures 4A** and **5A**). The frequency of CD45^+^ cells among total isolated polyp cells did not differ between treatment groups, suggesting that the extent of immune cell infiltration in polyps was not significantly affected (**Supplementary Figure 1D**). Expression of CD69 and Ki67 on CD4^+^ T cells was not altered, but there was an increased, although still relatively low, frequency of polyp CD4^+^ T cells expressing the TH2 associated marker ST2 after combination treatment (**Figure 4A**). Consistent with this, there was also an elevation of GATA3 expressing CD4^+^ cells in polyps after combination treatment, but there was no effect on RORγt expression (**Figure 4B**). However, there was an elevated expression of Tbet in a majority of CD4^+^ T cells in all organs after anti–PD-1 or combination treatment. This was accompanied with a markedly increased production of granzyme B, but not IFN-γ, by CD4^+^ T cells from all organs after *in vitro* stimulation (**Figure 4C**).

CD8^+^ T cells demonstrated a tendency of increased expression of CD69 and Ki67 in polyps after the combination treatment, however not significant in this data set (**Figure 5A**). Expression of Tbet and RORγt was not altered (data not shown). Production of IFN-γ or granzyme B after *in vitro* stimulation was not significantly altered after treatments, except for an increased production of IFN-γ in the spleen after combination treatment (**Figure 5B**). Taken together, the results indicate that the combination of α-GalCer and PD-1 blockade skewed CD4^+^ T cells toward a TH1 type response, while the effects on CD8^+^ T cells were more modest.

### Combination Treatment Increased Frequencies of GATA3-Expressing Treg Cells in Polyps and LP

**Figure 6:**
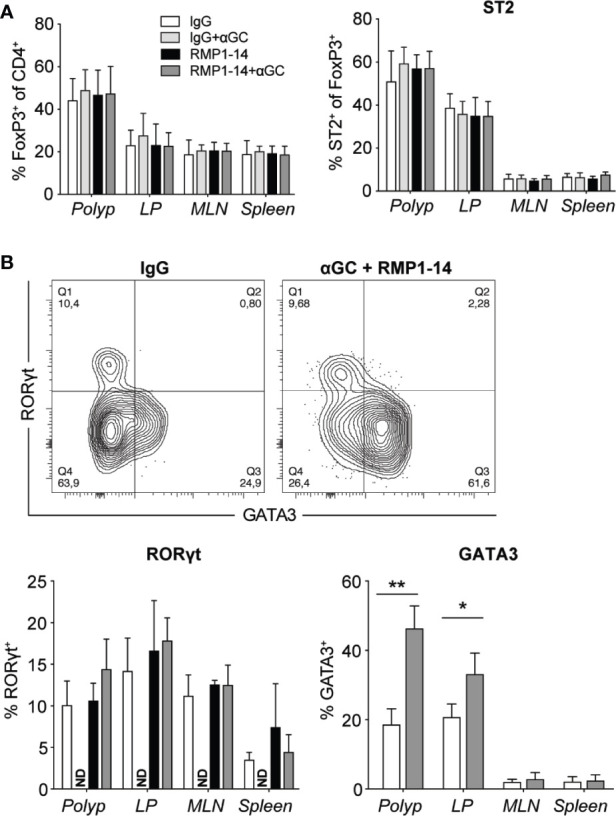
The combined treatment with αGC and anti-PD-1 blockade increased GATA3^+^ Treg cells. *Apc^Min/+^* mice were treated according to the treatment schedule, and polyp, polyp-free LP, MLN and spleen were harvested when the mice were sacrificed at 15 weeks of age. **(A)** Frequency of FoxP3^+^ Treg cells (CD4^+^FoxP3^+^) among total CD4^+^ cells (left), and the expression of ST2 on FoxP3^+^ Treg cells in treated *Apc^Min/+^* mice (right). **(B)** Representative staining of GATA3 and RORγt in FoxP3^+^ Treg cells and summary bar graphs are shown. Data are presented as mean ± SD of 5 to 8 mice per group. NT, not tested. Kruskal-Wallis test followed by Dunn’s multiple comparison post-test was used for statistical analyses. *p < 0.05, **p < 0.01.

Tumor immune infiltrates also contain immunoregulatory T cells (Treg) that play controversial role in CRC, as studies have reported both positive and negative correlations with patient outcome. This may relate to the abilities of Treg to suppress tumor promoting inflammation on the one hand, and to suppress tumor specific T cells on the other hand. These two functions have been suggested to associate with distinct populations of Treg cells with different suppressive abilities, that may reduce or increase tumor development, respectively (41, 42). In *Apc^Min/+^* mice, combination treatment, that reduced polyp development (**Figure 2**), did not affect Treg cell frequencies in polyps, and there was an unaltered expression level of FoxP3 and frequencies of ST2 (**Figure 6A**), CD69, Ki67 and KLRG1 positive Treg (data not shown). The expression of RORγt denotes a pathogenic subset of Treg cells in CRC tumors and *Apc^Min/+^* mouse polyps (41). We found no difference in RORγt expression in Treg cells of treated mice (**Figure 6B**), and Tbet levels remained unchanged (data not shown). However, the proportion of Treg cells expressing GATA3, a transcription factor important for the function of Treg cells in inflammatory conditions (43, 44), was increased in polyps and to a lesser extent in LP of combination treated mice (**Figure 6B**).

### Treatment With α-GalCer Alone or in Combination With Anti–PD-1 Enhanced M1-Like and Suppressed M2-Like Macrophages in the Spleen

**Figure 7:**
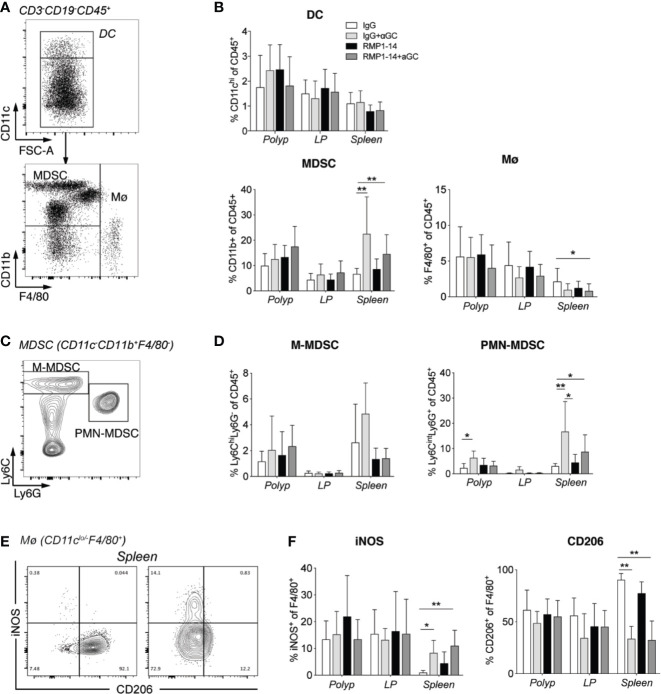
αGalCer induced PMN-MDSC and promoted a phenotypic M2 to M1 macrophage switch in the spleen. *Apc^Min/+^* mice were treated according to the treatment schedule and sacrificed at 15 weeks of age. Innate immune cells in polyps, polyp-free LP and spleen of treated mice were investigated by flow cytometry. **(A)** Gating strategy for dendritic cells (DC, CD3^−^CD19^−^CD45^+^CD11c^hi^), myeloid-derived suppressor cells (MDSCs) (CD3^−^CD19^−^CD45^+^CD11c^lo/neg^CD11b^+^), and macrophages (Mø, CD3^−^CD19^−^CD45^+^CD11c^lo/neg^F4/80^+^). **(B)** Frequency of DC, MDSC, and Mø among CD45^+^ cells in indicated tissues. **(C)** Gating strategy for M-MDSC (Ly6C^hi^Ly6G^+^CD11b^+^) and PMN-MDSC (Ly6C^int^Ly6G^+^CD11b^+^). **(D)** Frequency of M-MDSC and PMN-MDSC among CD45^+^ cells in indicated tissues. **(E)** Representative stainings of iNOS and CD206 on macrophages in spleen from control (left) and combination treated mice (right). **(F)** Frequencies of iNOS or CD206 expressing cells among macrophages in the different tissues. Data have been pooled from three independent experiments and are presented as mean ± SD of three mice per group. Kruskal-Wallis test followed by Dunn’s multiple comparison post-test was used for statistical analyses. *p < 0.05, **p < 0.01.

To further understand how α-GalCer synergized with PD-1 blockade to reduce polyps, we investigated innate immune cells in polyps, LP and spleen (**Figure 5**). There was no significant change in frequency of dendritic cells (DC) after either of the treatments, while macrophages were slightly reduced in spleen after combination treatment (**Figures 7A, B**). Whether combined with PD-1 blockade or not, α-GalCer treatment increased the frequency of macrophages expressing the M1 macrophage marker iNOS^+^ and decreased the frequency of macrophages positive for the M2 macrophage marker CD206^+^ in the spleen (**Figures 7E, F**) indicating a shift of macrophage phenotype from M2- toward M1-like. The frequencies of myeloid derived suppressor cells (MDSC) (**Figure 7B**), and specifically polymorphonuclear MDSC (PMN-MDSC, **Figures 7C, D**), were elevated in the spleen of *Apc^Min/+^* mice treated with α-GalCer alone or in combination with anti–PD-1. These data suggest that treatment with α-GalCer, regardless of anti–PD-1 blockade, altered the immune microenvironment in the spleen, indicated by the reduction of M2 macrophages and increase of M1 macrophages and PMN-MDSC.

## Discussion

Multiple studies in animal models have suggested that tumor immunotherapy using the iNKT cell ligand may be a promising approach to develop for clinical application. Animal studies and early clinical testing and have yielded encouraging results. α-GalCer mediated activation of iNKT cells leads to rapid production of a broad array of cytokines, including IFN-γ, TNF, IL-4, IL-13, IL-17, IL-21, and IL-22, and downstream activation of a multitude of immune cells. Activation of DC leads to upregulation of co-stimulatory molecules and IL-12 production and subsequent enhancement of T cell activation. Further, there is a bystander activation of cells such as NK cells and γδ T cells, altogether resulting in a broad activation of immune cells that contribute to tumor rejection (11). However, α-GalCer treatment results in massive iNKT cell activation that is followed by a loss of iNKT cells and long term anergy in remaining iNKT cells (33), and, thus, different approaches are being developed to prevent anergy induction and improve iNKT cell activation (8). PD-1 is often upregulated on T cells in the tumor environment, and the ligand PD-L1 is expressed on a multitude of tumor cells, as well as immune cells, leading to exhaustion and anergy of tumor infiltrating T cells after ligation of PD-1. We found PD-1 expression on polyp T cells and a striking upregulation of PD-1 on T cells in unaffected LP of *Apc^Min/+^* mice, suggesting that also the LP tissue surrounding polyps is highly affected by the presence of polyps, consistent with our previous studies (36). We therefore took the approach of using α-GalCer in combination with anti–PD-1 treatment, reasoning that PD-1 blockade could act at several levels and thereby improve the effects by α-GalCer. First, PD-1 blockade would prevent loss of iNKT cell anergy induction that follows from α-GalCer administration (33, 34, 45), which could lead to increased iNKT cell activation and enhanced TH1 tumor immunity in the *Apc^Min/+^* model. Second, PD-1 blockade would release conventional T cells from inhibition mediated by the high T cell expression of PD-1 induced by tumors, such as in LP and polyps in *Apc^Min/+^* mice. As expected, we show that addition of PD-1 antibody to α-GalCer treatment maintained iNKT cell levels, and further, increased proliferation of iNKT cells in all tissues, indicated by elevated Ki67 expression. Moreover, we find that the combined treatment shows synergistic effects in the suppression of both colonic and small intestinal polyps in *Apc^Min/+^* mice. This is consistent with previous findings that the PD-1/PD-L1 pathway imposed limitations on the antimetastatic effects of α-GalCer in the B16 melanoma transfer model (34, 45, 46). Here we have extended these findings and demonstrate that treatment with anti–PD-1 in combination with α-GalCer has strong suppressive effects on the development of spontaneous autochthonous intestinal tumors in a murine model that is based on a mutation found in a majority of CRC tumors. The adenomas in *Apc^Min/+^* mice represent an early stage in CRC development. *Apc^Min/+^* mice on a C57BL/6 genetic background die at a relatively early age (26), due to the development of numerous adenomas along the entire intestine, allowing too little time for adenomas to advance. However, when the *Apc^Min/+^* mutation is expressed in F1 hybrid mice, fewer adenomas develop and mice live longer (27, 31). These mice develop invasive adenocarcinomas, as well as a low frequency of lymph node metastases, firmly establishing *Apc^Min/+^* mice as a relevant model for early CRC.

We have previously shown that iNKT cells in *Apc^Min/+^* mice naturally exert a tumor promoting effect (36, 47). *Apc^Min/+^* mice deficient in iNKT cells had strongly reduced numbers and size of intestinal polyps. In polyps of untreated *Apc^Min/+^* mice, iNKT cells displayed a unique PLZF-negative phenotype (“iNKT-P^neg^”)associated with anti-inflammatory properties, and promoted an immune suppressive tumor microenvironment, characterized by increased Treg cells and M2-like macrophages (36). Interestingly, this is reminiscent of anti-inflammatory iNKT cells found in adipose tissue (47, 48). It was therefore somewhat surprising that α-GalCer treatment combined with PD-1 blockade strongly reduced polyp numbers. This is likely resulting from the demonstrated shift in the iNKT cell transcription factor expression in polyps from “iNKT-P^neg^” (PLZF^-^ Tbet^-)^ towards an iNKT1 pattern (PLZF^+^ Tbet^+^), a phenotype consistent with promotion of tumor immunity. This skewing of iNKT cell transcription factor expression was not seen in other organs, and was associated with a local upregulation of CD69, a marker of tissue residency, on polyp iNKT cells.

There was also a local upregulation in polyps and LP of the transcription factor GATA3 especially in Treg cells but to some extent also in conventional CD4 cells. In polyp Treg cells, GATA3 and RORγt were expressed in a non-overlapping manner, consistent with previous findings in different tissues (44). While RORγt^+^ Treg cells have been associated with tumor promotion in CRC and the *Apc^Min/+^* model (41), GATA3 expression is believed to generally reflect the activation status and function of Treg and limits their polarization to effector phenotypes, and enhances association of gut Treg with inflamed tissues (43, 44). Consistently, GATA3 regulates many genes in Treg cells (49), and thus, the increased expression of GATA3 is likely to result in altered function of Treg in in polyps after combination treatment.

Interestingly, PD-1 treatment was sufficient to induce significant changes in immune cells that are likely to contribute to enhanced tumor immunity. Both anti–PD-1 and combination treatment increased the proportion of iNKT cells with a TH1 like phenotype (iNKT1 cells) at the expense of “iNKT-P^neg^” cells specifically in polyps. Further, anti–PD-1 treatment was sufficient to induce a systemic increase in Tbet levels in CD4 T cells, while combination treatment resulted in elevated granzyme B expression after *in vitro* stimulation. This suggests that PD-1/combination treatment promotes CD4^+^ TH1 cells, a functional subset associated with increased tumor immunity. In the current study, however, we have not investigated putative tumor reactivity among these cells. Indeed, it was shown in CRC that increased expression of Tbet in tumor infiltrating T cells predicted increased patient survival (17). Restored expression of Tbet and a Tbet dependent increase in IFN-γ and CXCR3 expression was also found after combined check-point blockade (PD-1, CTLA-4, and LAG-3) in a murine leukemia model (50). Still, while anti–PD-1 treatment was sufficient to enhance Tbet expression in both polyp iNKT cells and systemically in CD4 T cells, this treatment only reduced the size of intestinal polyps, but not polyp numbers. α-GalCer treatment alone, on the other hand, resulted in changes in innate cells in the spleen. The splenic macrophage phenotype was shifted from a strong dominance of M2-like (80–90%) in control or anti–PD-1-treated mice, to around 30% M2-like macrophages in α-GalCer treated mice. This occurred whether α-GalCer was combined with anti–PD-1 treatment or not, and was paralleled with an increase of M1-like (iNOS^+^) macrophages. iNOS is known to be important for control of polyp growth in the *Apc^Min/+^* model, and infiltrating M1-like macrophages associate with better prognosis in CRC (51, 52). α-GalCer treatment also resulted in a reduction of PMN-MDSC in LP and spleen. However, these changes are not likely to have a significant effect on polyp immunity as they were not found in polyps and α-GalCer treatment alone did not significantly reduce tumor burden. In contrast, combined α-GalCer and anti–PD-1 treatment was required to achieve highly significant suppression of polyp development in both small intestine and colon, associated with a broad alteration of immune cells towards a more proinflammatory state generally associated with anti-tumor immunity. Taken together, this demonstrates that a combination of immune stimulation targeting iNKT cells and checkpoint blockade may be a promising approach to develop for improved tumor immunotherapy.

## Data Availability Statement

The raw data supporting the conclusions of this article will be made available by the authors, without undue reservation.

## Ethics Statement

The study was carried out in accordance with the national animal ethics regulations. The study was approved by the regional animal ethics board of Gothenburg (ethical permit number 1554/18).

## Author Contributions

SC conceived the study, planned experiments, analyzed data, and wrote the manuscript. YW planned and performed experiments, analyzed data, made figures, and wrote parts of the manuscript. MB planned and performed experiments, analyzed data, and critically reviewed the manuscript, HY provided essential reagents, contributed to experimental planning, and critically reviewed the manuscript. All authors contributed to the article and approved the submitted version.

## Funding

This work was supported by grants from the Swedish Cancer Foundation (18 0622) and the Swedish Research Council (2017-01821) to SC, grants from Adlerbertska Research Foundation and Wilhelm and Martina Lundgren Science Foundation to YW, and from Assar Gabrielsson Foundation to YW and MB. YW was financed by a PhD project grant to SC from the Sahlgrenska Academy, and a postdoc project grant from the Institute of Biomedicine, University of Gothenburg.

## Conflict of Interest

The authors declare that the research was conducted in the absence of any commercial or financial relationships that could be construed as a potential conflict of interest.

## Abbreviations

iNKT, invariant NKT; LP, lamina propria; MLN, mesenteric lymph node; NKT, natural killer T; i.p., intraperitoneal; TCR, T cell receptor; Treg, regulatory T cell; TH, T helper; α-GalCer, α-galactosylceremide.
