# Supplementary material for: Natural Killer T-Cell Agonist α-Galactosylceramide and PD-1 Blockade Synergize to Reduce Tumor Development in a Preclinical Model of Colon Cancer
Source: Front Immunol. 2020 Oct 20;11:581301. doi: 10.3389/fimmu.2020.581301 (PMC7606378; doi:10.3389/fimmu.2020.581301)
Supplement: Supplementary file 1 [file DataSheet_1.docx]

***Supplementary Figure 1***

**
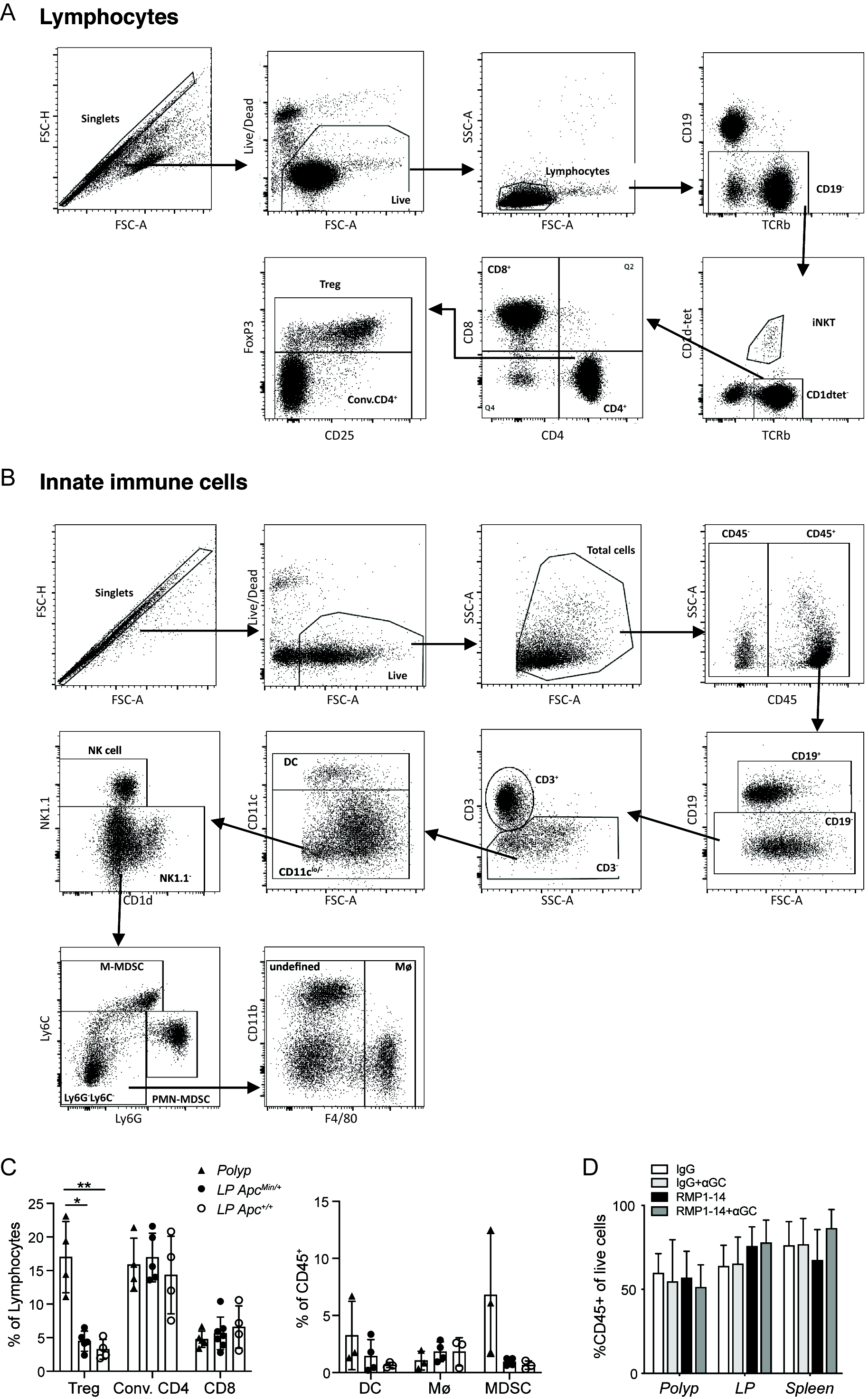
**

**Supplementary Figure 1. Gating strategies for immune cell identification.** Gating strategies are shown for spleen cells (A, B). (A) Lymphocyte populations gated for live singlet lymphocytes were defined according to surface marker and transcription factor expression: iNKT (CD19^-^TCRβ^+^CD1d-tetramer^+^), CD8^+^ T cells (CD19^-^TCRβ^+^CD1d-tetramer^-^CD8^+^), Treg (CD19^-^TCRβ^+^CD1d-tetramer^-^CD8^-^CD4^+^FoxP3^+^), conventional CD4^+^ (Conv. CD4^+^) T cells (CD19^-^TCRβ^+^CD1d-tetramer^-^CD8^-^CD4^+^FoxP3^-^) (B) Gating strategy for innate immune cell populations among singlet live total cells: dendritic cells (DC, CD45^+^ CD19^-^CD3^-^CD11c^hi^), natural killer cells (NK, CD45^+^ CD19^-^CD3^-^CD11c^lo/neg^NK1.1^+^), monocytic myeloid-derived suppressor cells (M-MDSC, CD45^+^ CD19^-^CD3^-^CD11c^lo/neg^NK1.1^-^Ly6C^hi^Ly6G^-^), polymorphonuclear myeloid-derived suppressor cells (PMN-MDSC, CD45^+^ CD19^-^CD3^-^CD11c^lo/neg^NK1.1^-^Ly6C^int^Ly6G^+^) and macrophages (Mø, CD45^+^ CD19^-^CD3^-^CD11c^lo/neg^NK1.1^-^Ly6C^-^Ly6G^-^F4/80^+^). (C) The representation of T cells and innate immune cells in polyp and unaffected lamina propria (LP) tissues in untreated *Apc^Min/+^* and *Apc^+/+^* mice. Data are presented as mean ± SD of 3 – 5 mice. (D) Frequencies of CD45^+^ cells among live total cells (gated as in Supplementary Figure 1B) in indicated organs of the different treatment groups. Data are presented as mean ± SD of 9-21 mice.

***Supplementary Figure 2***

**
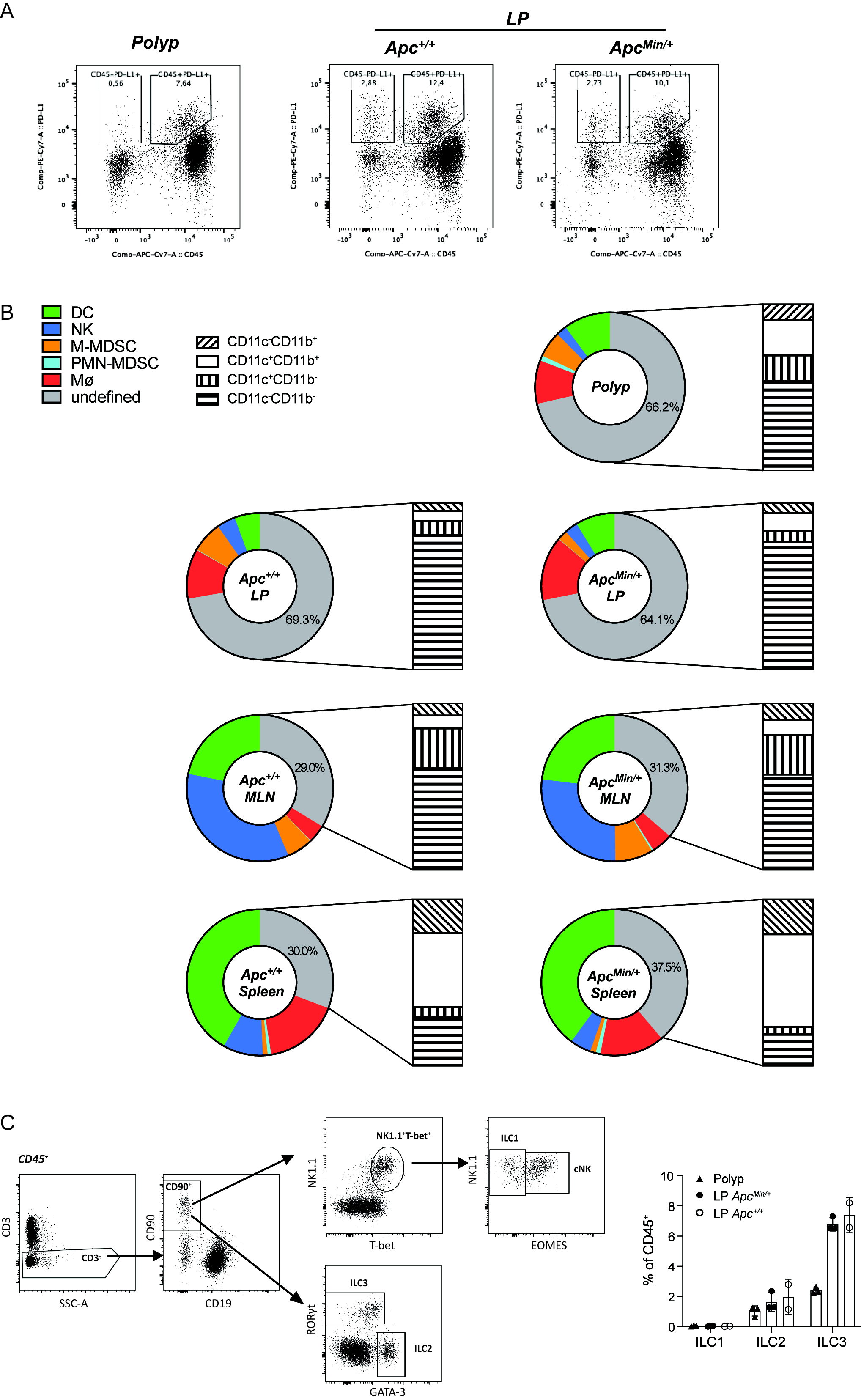
**

**
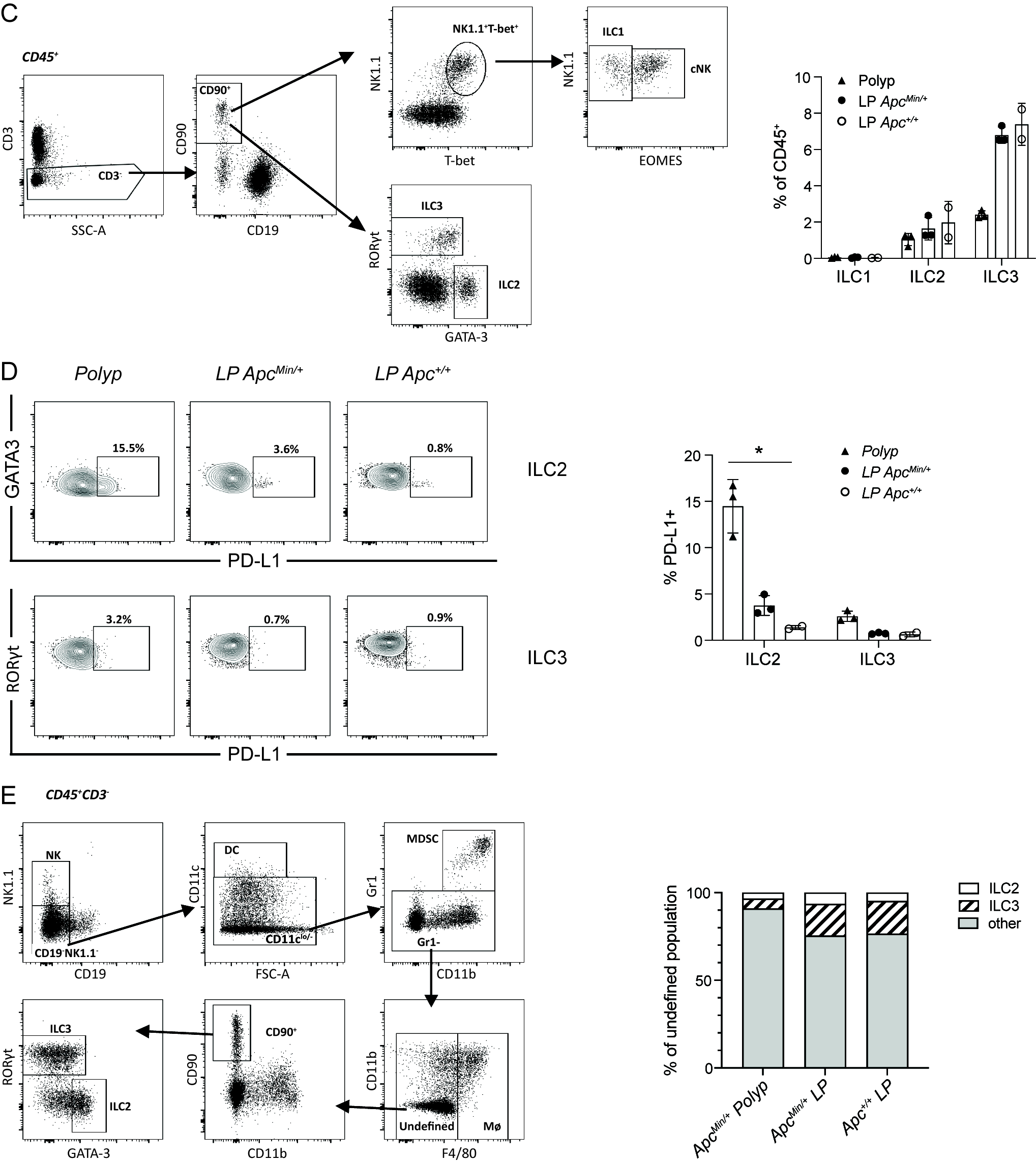
**

**Supplementary Figure 2. PD-L1 expression in different organs of *Apc^Min/+^* and *Apc^+/+^* mice.** (A) Representative stainings of PD-L1 expression on CD45^+^ and CD45^-^ cells among live total cells in polyp and unaffected lamina propria (LP). (B) Pie charts show the proportions of different populations among CD45^+^ PD-L1^+^ innate immune cells (gated as in Supplemental Figure 2A) in the indicated organs. Bar graphs show the expression of CD11c and CD11b on subpopulations of “undefined” cells (as gated in Supplementary Figure 1B). (C) Gating strategy for innate lymphoid cells (ILC) subsets (shown for cells from MLN, dot plots to the left). The same strategy was followed to analyze intestinal tissues. CD45^+^CD3^-^CD19^-^CD90^+^ cells were gated, and among these, ILC1 were defined as NK1.1^+^T-bet^+^EOMES^-^; ILC2 were defined as GATA3^+^; and ILC3 were defined as RORγt^+^. The bar graph (right panel) shows the frequency of ILC subsets in polyps and LP in *Apc^Min/+^* and *Apc^+/+^* mice. (D) Representative stainings (left panels) and summary plot (right panel) of PD-L1 expression on ILC2 and ILC3 in polyps and LP in *Apc^Min/+^* and *Apc^+/+^* mice. (E) Gating strategy to analyze the contribution of ILC to the “undefined” population (left panel) and the bar graph indicating the proportions of ILC2 and ILC3 within the “undefined” population (right panel). (C-E) Data are presented as the mean of 3 mice for *Apc^Min/+^* polyp and LP, and 2 mice for *Apc^+/+^* LP. Kruskal-Wallis test followed by Dunn’s multiple comparison post-test was used for statistical analyses. * p<0.05.
